# System Usability Scale Benchmarking for Digital Health Apps: Meta-analysis

**DOI:** 10.2196/37290

**Published:** 2022-08-18

**Authors:** Maciej Hyzy, Raymond Bond, Maurice Mulvenna, Lu Bai, Alan Dix, Simon Leigh, Sophie Hunt

**Affiliations:** 1 School of Computing Ulster University Newtownabbey United Kingdom; 2 Organisation for the Review of Care and Health Applications Daresbury United Kingdom; 3 Computational Foundry Swansea University Swansea United Kingdom; 4 Institute of Digital Healthcare University of Warwick Coventry United Kingdom

**Keywords:** mHealth SUS scores meta-analysis, SUS for digital health, digital health apps usability, mHealth usability, SUS meta-analysis, mHealth, mobile app, mobile health, digital health, System Usability Scale

## Abstract

**Background:**

The System Usability Scale (SUS) is a widely used scale that has been used to quantify the usability of many software and hardware products. However, the SUS was not specifically designed to evaluate mobile apps, or in particular digital health apps (DHAs).

**Objective:**

The aim of this study was to examine whether the widely used SUS distribution for benchmarking (mean 68, SD 12.5) can be used to reliably assess the usability of DHAs.

**Methods:**

A search of the literature was performed using the ACM Digital Library, IEEE Xplore, CORE, PubMed, and Google Scholar databases to identify SUS scores related to the usability of DHAs for meta-analysis. This study included papers that published the SUS scores of the evaluated DHAs from 2011 to 2021 to get a 10-year representation. In total, 117 SUS scores for 114 DHAs were identified. R Studio and the R programming language were used to model the DHA SUS distribution, with a 1-sample, 2-tailed *t* test used to compare this distribution with the standard SUS distribution.

**Results:**

The mean SUS score when all the collected apps were included was 76.64 (SD 15.12); however, this distribution exhibited asymmetrical skewness (–0.52) and was not normally distributed according to Shapiro-Wilk test (*P*=.002). The mean SUS score for “physical activity” apps was 83.28 (SD 12.39) and drove the skewness. Hence, the mean SUS score for all collected apps excluding “physical activity” apps was 68.05 (SD 14.05). A 1-sample, 2-tailed *t* test indicated that this health app SUS distribution was not statistically significantly different from the standard SUS distribution (*P*=.98).

**Conclusions:**

This study concludes that the SUS and the widely accepted benchmark of a mean SUS score of 68 (SD 12.5) are suitable for evaluating the usability of DHAs. We speculate as to why physical activity apps received higher SUS scores than expected. A template for reporting mean SUS scores to facilitate meta-analysis is proposed, together with future work that could be done to further examine the SUS benchmark scores for DHAs.

## Introduction

According to Nielsen [1], “usability is a quality attribute that assesses how easy user interfaces are to use. The word ‘usability’ also refers to methods for improving ease-of-use during the design process.” In Nielsen’s [1] model, usability consists of a number of components, including the system’s learnability, efficiency, memorability, errors, and satisfaction.

According to the International Organization for Standardization, “usability is the extent to which a product can be used by specified users to achieve specified goals with effectiveness, efficiency, and satisfaction in a specified context of use” [2].

The public is increasingly searching for digital health apps (DHAs) in app stores to help self-manage their health and well-being [3]. With the uptake of DHAs, national health care organizations such as the National Health Service in the United Kingdom are offering curated access to health care apps as part of social prescription and related services [4].

The usability of DHAs is important as inferior usability could negatively impact the adoption of such technologies, and potentially, their users’ health [5]. For example, a study conducted in 2019 found that self-management DHAs with higher rated usability (rated based on heuristic usability testing) lead to increased exercise engagement and quality of life in patients with breast cancer [6]. Reliably measuring the usability of DHAs can be used to distinguish between usable and less usable DHAs and help identify DHAs that may require improved usability.

The System Usability Scale (SUS), commonly described as a “quick and dirty” way of measuring usability, is a short 10-item questionnaire (each question with a Likert scale ranging from strongly agree to strongly disagree) designed to measure the usability of a system [7]. The SUS is a well-designed, balanced survey consisting of 5 questions with positive statements and 5 questions with negative statements, with scores ranging from 0 to 100. The current literature suggests that a score of 68 is a useful benchmark (mean SUS score), where 50% of apps fall below and above it [8]. Sauro and Lewis [8] discuss using data from 446 studies and 5000 individual SUS responses that indicate a mean SUS score of 68 (SD 12.5) [8]. Hence, the standard normal SUS distribution is said to be 68 (SD 12.5).

The SUS has become a common method for measuring the usability for different digital products or systems (including DHAs) since its development in 1986 [9]. According to a scoping review from 2019 [10], SUS was the most frequently used questionnaire for evaluating the usability of DHAs. However, the normal SUS distribution evaluated by Sauro and Lewis [8] (68 SD 12.5) was not likely representative of SUS scores achieved by mobile apps or DHAs.

The mHealth App Usability Questionnaire (MAUQ) is a validated alternative to SUS for measuring usability that is tailored to mobile health (mHealth) apps [10]. Although MAUQ may be more suitable for measuring the usability of DHAs, it is a relatively new scale developed in 2019. SUS has been used to evaluate DHAs since their inception; however, it remains to be seen whether the mean 68 (SD 12.5) benchmarking distribution represents the SUS scores achieved by DHAs.

The aim of this study was to determine if the widely accepted benchmark and SUS distribution of mean 68 (SD 12.5) is reliable for evaluating the usability of DHAs. This work is important given that the SUS benchmarking distribution that is being used is assumed to represent the usability of DHAs even though this standard SUS distribution was developed based on the usability of systems more generally (well beyond the genre of DHAs). Given that SUS is a frequently used tool for measuring the usability of DHAs, this study is needed to reassure researchers if the mean 68 (SD 12.5) distribution benchmark is reliable when evaluating DHAs using SUS and discover if a different SUS benchmark should be used for different genres of DHAs. To determine these findings, a comparison of published SUS scores from evaluated DHAs with the standard SUS distribution was conducted.

## Methods

### SUS Score

A SUS score is computed using the 10 Likert ratings that is typically completed by a user after having been exposed to the system for a period of time. The process for computing a SUS score is as follows:

1. Subtract 1 from the user’s Likert ratings for odd-numbered items or questions.
2. Subtract the user’s Likert ratings from 5 for even-numbered items.
3. Each item score will range from 0 to 4.
4. Sum the numbers and multiply the total by 2.5.
5. This calculation will provide a range of possible SUS scores from 0 to 100 [7].

### Data Collection

Table 1 provides the criteria and search strategy for selecting the research papers that were used to conduct the meta-analysis on SUS scores. In this study, we aimed to collect papers that published the SUS scores of the evaluated DHAs after 2011. This criterion allowed us to curate a relatively “modern” set of SUS scores from DHA evaluations with a 10-year representation. A total of 114 DHAs producing 117 SUS scores were collected to conduct this meta-analysis.

Table 2 provides the number of papers and SUS scores that were used in this study to populate a DHA SUS data set.

**Table 1:** Population, Intervention, Comparator, Outcome, and Study Design framework for the data collection of digital health app (DHA) System Usability Scale (SUS) scores.

Frame	Inclusion criterion	Exclusion criterion
Population	Members of the general population—globally	Developers or designers of DHA that conducted SUS on their own product
Intervention	DHA	Not a DHA and research papers published before 2011
Comparator	N/A^a^	N/A
Outcome	SUS score or mean SUS score for DHA	SUS score not conducted by end users
Study design	The data set of SUS scores for measuring the usability of DHAs was collected using 5 search engines: ACM Digital Library, IEEE Xplore, CORE, PubMed, and Google Scholar. The keywords and queries used in the search included: “health app SUS,” “mhealth SUS,” “digital health apps SUS,” “mobile health SUS,” “mhealth apps usability,” and “mental health apps SUS.”	N/A

^a^N/A: not applicable.

**Table 2:** Number of papers and System Usability Scale (SUS) scores per year.

Year	Paper (N=19), n (%)	SUS score (N=117), n (%)
2014	2 (11)	14 (12)
2015	2 (11)	2 (1.7)
2016	2 (11)	3 (2.6)
2017	1 (5)	2 (1.7)
2018	3 (16)	71 (60.1)
2019	3 (16)	9 (7.7)
2020	3 (16)	12 (10.2)
2021	3 (16)	4 (3.4)

### Study Screening

The research papers included in this study were screened by title and abstract. If the research paper included a SUS score for a DHA and the SUS evaluation was conducted by end users, it was included in this study.

### Risk of Bias

SUS is a simple method of measuring the usability of hardware and software that should be conducted by end users. When conducting this study, the exclusion criterion was set to not include SUS evaluation scores that were provided by the developers or designers of the DHA, due to potential bias. However, none of the SUS scores collected met that exclusion criterion.

There may also be a bias if there are more SUS scores published for DHAs of a particular genre, or there could be a publication bias, as researchers are more likely to publish studies that achieved “good” (above the 68 benchmark) SUS scores. This is related to the file drawer effect [11], where researchers withhold studies that show nonsignificant or negative results (*P*>.05). Literature indicates that about 95% of studies in the file drawer contain nonsignificant results, whereas journals contain a disproportionate number of studies with type 1 errors.

When there are more SUS scores published for DHAs of a particular genre, they could be overrepresented in a general health app SUS distribution and perhaps skew the distribution. This bias could be avoided by conducting this study on a data set where the different genres of DHAs are balanced. Publication bias could be countered by collecting new data sets where end users complete SUSs when viewing a large random sample of DHAs.

SUS has been developed in English to be used by English-speaking users. Using SUS with non-English speakers requires a new version of SUS that needs to be adapted and validated. Otherwise, there could be language and cultural bias in the assessment. Cross-cultural adaptation guidelines [12] could be used to adapt SUS; previously, these guidelines have been used to develop the Indonesian version of SUS [13]. Moreover, a study conducted in 2020 examined the Arabic, Chinese, French, German, and Spanish versions of the SUS [14]. The study found that these SUS versions were adequately adapted; however, cultural differences had to be highlighted [14]. Furthermore, the different devices and genres of DHAs may need their own, more specific SUS benchmarks.

### Data Extraction

The study-specific data that were extracted from the research papers included first author’s name, DHA’s focused health area, DHA’s name, device that the DHA was used on, platform the DHA is available on, sample size used to calculate the mean DHA SUS score, year the research paper was published in, and DHA SUS score.

### Data Analysis

The data were separated into 3 subsets: (1) a SUS distribution including all DHAs, (2) a SUS distribution with only SUS scores from physical activity apps, and (3) a SUS distribution including all apps except physical activity apps. This separation was done due to the large frequency of physical activity apps that are present in the data set and the high mean of these apps (83.28, SD 12.39), which dominated the shape of the probability distribution.

R statistical software (version 4.0.3; R Foundation for Statistical Computing) was used to conduct the meta-analysis, compute statistics, and produce graphs. Shapiro-Wilk normality tests were used to test whether the SUS distributions were normally distributed (where *P*<.05 denotes that the distribution is not normal). Skewness and kurtosis were computed to determine how symmetrical (or unsymmetrical) and heavy- or light-tailed the data distributions are. The data were also visually explored using density plots, histograms, and boxplots to interrogate the distribution of SUS scores.

Wilcoxon signed rank tests and 1-sample, 2 tailed *t* tests were used to compare the mean SUS scores of DHAs with the widely accepted SUS distribution (mean 68, SD 12.5) that is typically used for benchmarking usability. *p* values <.05 were considered statistically significant in this study.

## Results

Table 3 provides the mean, SD, and frequency of DHAs for each category. The “physical activity” category mainly included fitness apps. The “health care” category included DHAs that help with self-managing health and well-being, including living with and the treatment of obesity, allergies, suicide prevention, depression, and smoking cessation. The category “first aid, CPR, and choking” mainly included DHAs that assist with first aid and cardiopulmonary resuscitation. The category “diet, food, and nutrition” included diet apps and food and nutrition apps. The category “health information” included DHAs that provide health-related information and educational content. See Multimedia Appendix 1 [5,15-32] for more information.

Table 4 provides a summary of the characteristics of the 3 SUS distributions: (1) a SUS distribution from all categories of DHAs, (2) a SUS distribution from physical activity apps only, and (3) a SUS distribution from all categories excluding the physical activity apps. It is clear that the SUS distributions from all DHAs and the SUS distribution from physical activity apps only are not normally distributed. However, the distribution of SUS scores from all DHAs excluding physical activity apps is more akin to a normal distribution. The participant sample sizes used to collect the SUS scores have distribution of 6 (SD 6.16; range 2-31). See Multimedia Appendix 1 for the sample size of each SUS score collected.

Table 5 provides a summary of the 1-sample, 2-tailed *t* tests. The table indicates that the SUS distribution from all DHAs and the SUS distribution from physical activity apps only are statistically different distributions compared to the accepted mean 68 (SD 12.5) SUS distribution (*P*=.002). However, when excluding physical activity apps, the 1-sample, 2-tailed *t* test suggests that the distribution is comparable to the standard SUS distribution of mean 68 (SD 12.5).

**Table 3:** Category and frequency of apps included in this study.

Category	App (N=117), n (%)	SUS^a^ score, mean (SD)
Physical activity	66 (56.4)	83.28 (12.39)
Health care	25 (21.4)	71.30 (12.72)
First aid, CPR^b^, and choking	16 (13.7)	61.29 (15.08)
Diet, food, and nutrition	8 (6.8)	71.06 (14.55)
Health information	2 (1.7)	69.45 (5.30)

^a^SUS: System Usability Scale.

^b^CPR: cardiopulmonary resuscitation.

**Table 4:** Characteristics of System Usability Scale (SUS) probability distributions for the 3 categories.

Characteristic	SUS scores from all categories	SUS scores from all categories excluding physical activity apps	SUS scores from physical activity apps only
*P* value (Shapiro-Wilk)	.002	.24	.001
Mean (SD)	76.64 (15.12)	68.05 (14.05)	83.28 (12.39)
Median	78.75	68.30	86.00
Skewness	–0.52	–0.39	–0.69
Kurtosis	2.67	2.74	2.55
Standard error	1.4	1.97	1.53

**Table 5:** Results from hypothesis test.

Hypothesis, test		*P* value	95% CI
**All categories versus standard SUS^a^ distribution**			
	1-sample, 2-tailed *t* test	<.001	73.87-79.41
	Wilcoxon signed rank test with continuity correction	<.001	74.50-80.00
**All categories excluding physical activity apps versus standard SUS distribution**			
	1-sample, 2-tailed *t* test	.98	64.10-72.00
	Wilcoxon signed rank test with continuity correction	.86	64.30-72.60
**Physical activity apps only versus standard SUS distribution**			
	1-sample, 2-tailed *t* test	<.001	80.23-86.33
	Wilcoxon signed rank test with continuity correction	<.001	80.50-87.50

^a^SUS: System Usability Scale.

**Figure 1:**
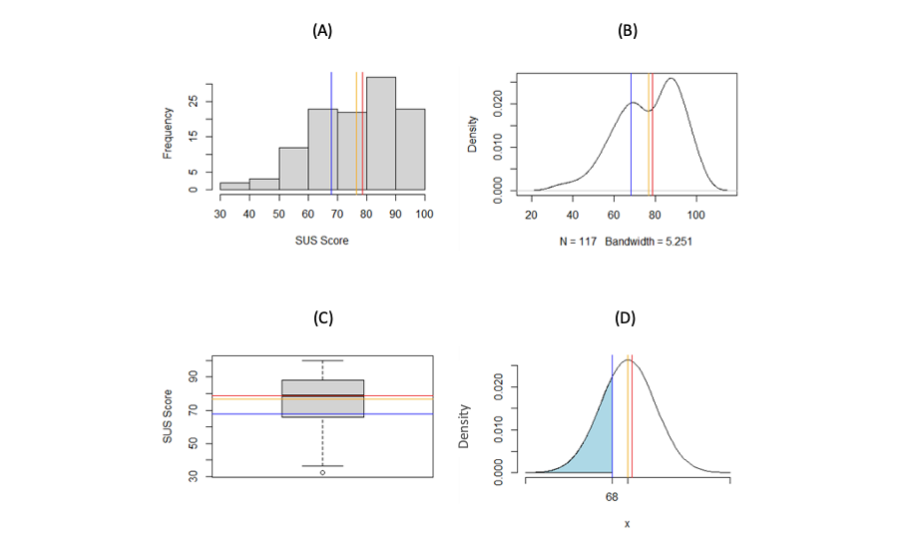
Analysis of SUS distribution for all categories of digital health apps: A) histogram of SUS scores, B) density plot of SUS scores, C) boxplot of SUS scores, and D) normal curve probabilities of SUS scores for all categories (mean 76.64, SD 15.12; shaded area: 0.2839). Blue line=68 (average SUS score for apps), red line=78.75 (median), orange line=76.64 (mean). SUS: System Usability Score.

The graphs in Figure 1 show that there is an unexpected peak in SUS scores for the range of 80-90, and the frequency in this range is greater than that for the range of 60-70. Table 3 shows the frequency of SUS scores for each category and indicates that the physical activity category has the highest frequency, which could be responsible for the peak in the 80-90 SUS score range/bin.

**Figure 2:**
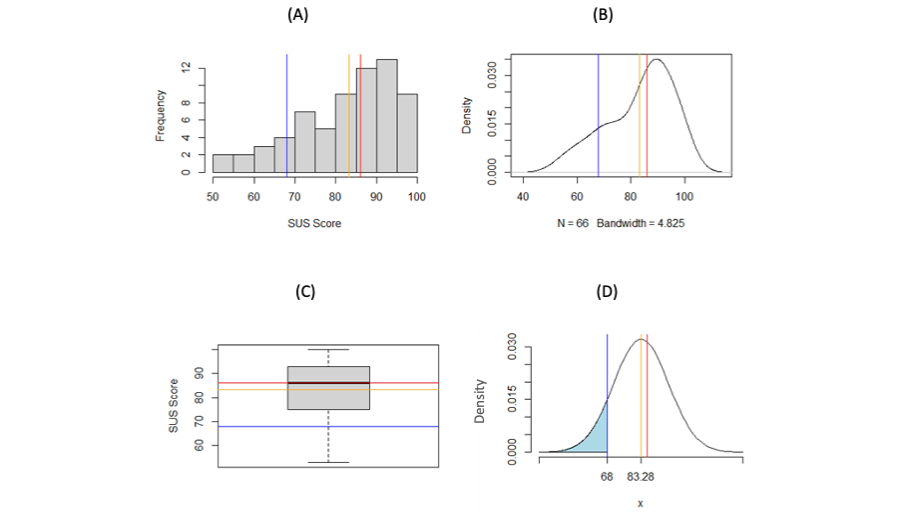
Analysis of SUS distribution for physical activity apps only: A) histogram of SUS scores, B) density plot of SUS scores, C) boxplot of SUS scores, and D) normal curve probabilities of SUS scores for all categories (mean 83.28, SD 12.39; shaded area: 0.1088). Blue line=68 (average SUS score for apps), red line=86 (median), orange line=83.28 (mean). SUS: System Usability Score.

**Figure 3:**
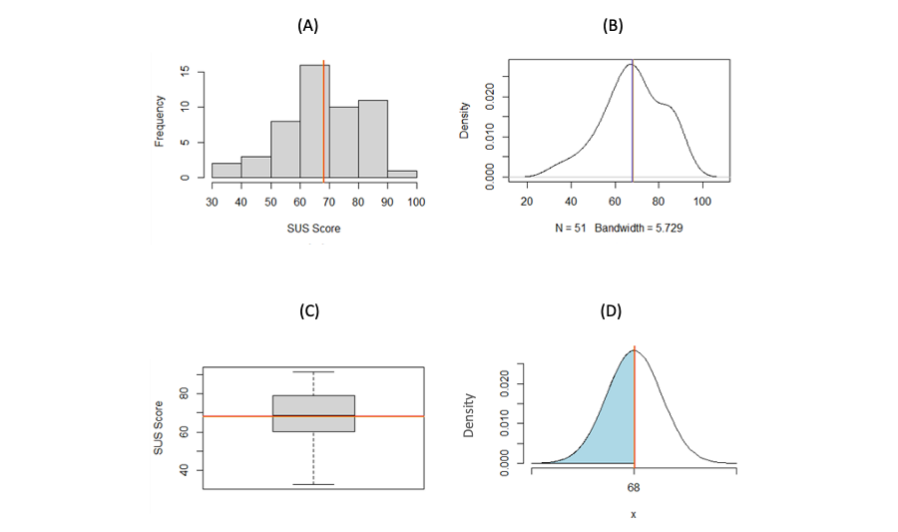
Analysis of SUS distribution for all categories excluding physical activity apps: A) histogram of SUS scores, B) density plot of SUS scores, C) boxplot of SUS scores, and D) normal curve probabilities of SUS scores for all categories (mean 68.05, SD 14.05; shaded area: 0.4985). Blue line=68 (average SUS score for apps), red line=68.30 (median), orange line=68.05 (mean). SUS: System Usability Score.

Figure 1 visually demonstrates that the SUS distribution for all DHAs is asymmetrical. For example, when all categories are included, the cumulative distribution function indicates that there is a 28.39% probability that the SUS score will be 68 or less, whereas the accepted standard probability is 50% that the SUS score will be 68 or less [8]. Figure 2 indicates that physical activity apps are responsible for the second “peak” in Figure 2A and B. The mean of 83.28 is much greater than the expected mean of 68. The SUS scores for physical activity apps could be inflated or that these apps typically have a greater degree of usability, which would need to be determined by conducting further studies. Figure 2 shows that there is a probability of 10.88% that the SUS score in the category of physical activity will be 68 or less, indicating that this distribution is very different compared to the expected SUS distribution of mean 68 (SD 12.5). Figure 3 shows that the mean and median are both very close to 68 after removing SUS scores from physical activity apps. This finding helps confirm that the SUS score distribution of DHAs is similar to that of the accepted standard SUS distribution. When using this distribution, Figure 3D shows that there is a probability of 49.85% that the SUS score will be 68 or less, making it very similar to the standard.

## Discussion

### Principal Findings

The data set used for this study contained 117 SUS scores collected from 114 DHAs (some apps were assessed by different end users, such as clinicians, researchers, or participants, that gave them different SUS scores, which were included in this study). The SUS mean when all of the apps are included is 76.64; however, this mean score lies between 2 peaks, as seen in Figure 1B. Thus, this mean may not be suitable for benchmarking DHAs. In Figure 1B, the blue line indicates the mean SUS score of 68 when all SUS scores are included in the distribution, which is exactly in line with the first peak in the distribution. This finding indicates that many of the DHAs follow a similar SUS distribution to that in the expected standard.

When investigating the results in Figure 1, we explored the cause of the second peak in Figure 1B. Hence, due to frequency of physical activity apps (66 DHAs) in the data set and the mean of 83.28 (SD 12.39; Table 3), a distribution of only physical activity apps was examined (Figure 2). We discovered that the second peak in Figure 1B was driven by the SUS scores of physical activity apps.

When the SUS scores of physical activity apps are excluded from the data set, the SUS score distribution for DHAs become normally distributed (mean 68.05, SD 14.05) and is similar to the widely used SUS distribution (mean 68, SD 12.5). Although the SUS distribution of DHAs have a slightly greater SD (14.05 vs 12.5), this finding could be due to the small sample size in this study. The results indicate that the standard SUS score benchmark of 68 can be used when evaluating DHAs. This assumption was important to test given that the accepted distribution of mean 68 (SD 12.5) was not primarily based on SUS scores from mobile apps, or in particular DHAs. The usability of systems may generally improve over time, which could change the average SUS score that would be achieved by digital systems. Moreover, given that DHAs can be critically important apps to users (nonrecreational or nonhedonic), their usability could be greater, hence achieving higher SUS scores.

The paper that published the SUS scores of these 65 physical activity apps focused on the most popular apps available to conduct their SUS evaluation, which could indicate that more popular apps are perhaps more usable. Further research is needed to determine if there is a link between app popularity and the usability of DHAs. Other possibilities are inflated SUS scores, popularity in the market [33] leading to better usability, and greater budgets to invest into usability. More familiar design has been shown to influence usability, as stated by Jakob’s law: “users spend most of their time on other sites. This means that users prefer your site to work the same way as all the other sites they already know” [34].

Developers of physical activity apps appear to be investing a lot into usability. For example, to encourage physical activity for those with low socioeconomic status and youths, the prototyping for a smartphone user-centric framework for developing game-based physical activity apps has been created [35]. A study from 2017, where the top 50 health and fitness apps were downloaded from the Apple app store, found that physical activity and weight loss apps most frequently (97%) used gamification [36]. Gamification has been shown to improve the use of physical activity apps [37], which could explain the higher-than-expected usability of physical activity apps and indicates that a different benchmark may need to be used when dealing with physical activity apps.

### Set of Guidelines for Presenting SUS Analysis to Facilitate High-Quality Meta-analyses

When conducting the meta-analysis for this paper, we encountered a couple of problems when gathering the SUS scores from research papers. Some papers used the word “expert” when stating the sample size of reviewers who used SUS to assess a DHA. It was unclear as to whether the word “expert” referred to an expert usability reviewer or expert in the health area for which a DHA has been developed. Clearly stating who the reviewer is would be useful when conducting a rigorous meta-analysis for SUS.

Textbox 1 recommends a standard template for reporting SUS analysis and scores that could be helpful when presenting an SUS analysis to facilitate high-quality meta-analyses.

**Recommended template for reporting mean System Usability Scale (SUS) scores to facilitate meta-analyses.**

**Participants**

- Novice users (those with no experience in using the system being assessed)
- Expert users (those who already have experience in using the system)
- Expert user-experience evaluators
- Representative users (those who are likely to use the app; eg, recruiting doctors when testing a medical system) and nonrepresentative users (anyone outside the domain of interest; eg, recruiting any person to test the usability of a fitness app)

**Context**

- include information such as a usability testing session with prescribed tasks, a usability testing session without prescribed tasks, SUS scores collected after a trial (lasting n days, weeks, or months), or other details (eg, remote usability test and lab-based or in-situ [eg, workplace or “in the wild”])

Sample size (n)

Mean (SD) score (rounded to 2 decimal places)

Median score (min/max; rounded to 2 decimal places)

Standard error of the mean (rounded to 2 decimal places)

95% CI (lower to upper)

Test (eg, 1-sample, 2-tailed *t* test)

SUS grade (A-F)

### Related and Future Works

Although this study assessed SUS for evaluating DHAs, there are other scales that could be used, which includes the previously mentioned MAUQ. Currently, there are 4 versions of the MAUQ, 2 for stand-alone apps (provider and patient versions) and 2 for interactive mHealth apps (provider and patient versions). The SUS and MAUQ are correlated, but the correlation is not strong (*r*=0.6425) [38].

A systematic literature review [39] evaluated the methodologies of usability analyses, domains of usability being assessed, and results of usability analyses. The paper concluded that out of the 3 usability domains in MAUQ, only satisfaction is regularly assessed. A similar meta-analysis to the one conducted in this study could be done with the MAUQ.

The usability of DHAs can be improved; in the study by Liew et al [40], researchers provided insight and suggestions for improving the usability of health and wellness mobile apps. The paper concluded that better connectivity between mHealth suppliers and users will have a positive outcome for the mHealth app ecosystem and increase the uptake of mHealth apps.

Improving usability is important as the lack of it can slow down the adoption of DHAs. Islam et al [5] investigated the usability of mHealth apps in Bangladesh using a heuristic evaluation and the SUS. The paper concluded that the usability of DHAs in Bangladesh is not satisfactory and could be a barrier for the wider adoption of DHAs.

As the SUS scores for physical activity apps were higher than other apps in this study, future work is needed to explore how these scores could be inflated or whether these apps have a greater degree of usability.

The study conducted in this paper could be expanded in the following ways. Future studies could be done by comparing the SUS scores evaluated by experts and nonexperts. The meta-analysis conducted here could be repeated on a bigger data set. A SUS meta-analysis could be conducted for a wide range of health app categories to validate if all follow the standard SUS distribution (mean 68, SD 12.5). A study with randomly selected apps could be conducted with several recruited end users completing the SUS questionnaire that would allow for a more unbiased distribution of SUS scores.

The paper with 65 physical activity apps [15] focused specifically on the most popular apps. Research could be done to determine if there is a link between popularity and the usability of DHAs when using the SUS or MAUQ.

### Limitations

This study has a few limitations. This meta-analysis collected SUS results from 19 papers—some of which used a mean SUS score resulting from as few as 2 or 3 reviewers. Some of the reviewers could have been “generous” when filling the SUS questionnaire, resulting in inflated SUS scores. The data set used for this study is small (SUS scores: n=117). Moreover, 65 of the physical activity apps used in this study came from the same paper [15]. This paper used 2 reviewers when evaluating each of the apps. A speculation can be made that since 65 physical activity apps were being evaluated, it is possible that the reviewers had limited time to spend on each of the app evaluations, although no information is provided to support this.

This study was conducted in 2021, and some of the apps may have been updated. Various changes to the design could have been made since their SUS score was evaluated, and thus, the SUS score may no longer be applicable to the app.

### Conclusion

The aim of this study was to conduct a meta-analysis to determine if the standard SUS distribution (mean 68, SD 12.5) for benchmarking is applicable to evaluating DHAs. This study compared the standard SUS score distribution to the distribution for different categories of DHAs. The data for this study were collected from different research papers that were found using different search engines or research repositories. This study indicates that the SUS distribution of DHAs (when excluding physical activity apps) is similar to the widely used SUS distribution. This work implies that the SUS and existing benchmarking approaches could be used to evaluate DHAs and that the SUS could be used by health care departments and organizations such as the National Health Service or Organisation for the Review of Care and Health Applications to validate and assure the quality of DHAs in terms of their usability. Readers of this work may also choose to use our SUS distribution (mean 68.05, SD 14.05) for benchmarking the SUS scores of DHAs.

## Appendix

Multimedia Appendix 1 Collected System Usability Scale scores.

## Abbreviations

DHA: digital health app
MAUQ: mHealth App Usability Questionnaire
mHealth: mobile health
SUS: System Usability Scale
