# Supplementary material for: System Usability Scale Benchmarking for Digital Health Apps: Meta-analysis
Source: JMIR Mhealth Uhealth. 2022 Aug 18;10(8):e37290. doi: 10.2196/37290 (PMC9437782; doi:10.2196/37290)
Supplement: Multimedia Appendix 1 [file mhealth_v10i8e37290_app1.docx]

| **Reference and first author** | **Focused health area** | **Name** | **Device** | **Available** | **Sample size** | **Year** | **SUS score** |
| --- | --- | --- | --- | --- | --- | --- | --- |
| [16]  Ferrara et al., | Diet, food & nutrition | FatSecret | Mobile Phone | iTunes/GP | 3 undergraduate students | 2019 | 63.3 |
| [16]  Ferrara et al., | Diet, food & nutrition | LifeSum | Mobile Phone | iTunes/GP | 3 undergraduate students | 2019 | 89.2 |
| [16]  Ferrara et al., | Diet, food & nutrition | MyPlate | Mobile Phone | iTunes/GP | 3 undergraduate students | 2019 | 86.7 |
| [16]  Ferrara et al., | Diet, food & nutrition | Argus | Mobile Phone | iTunes/GP | 3 undergraduate students | 2019 | 70 |
| [16]  Ferrara et al., | Diet, food & nutrition | Lose It! | Mobile Phone | iTunes/GP | 3 undergraduate students | 2019 | 59.2 |
| [16]  Ferrara et al., | Diet, food & nutrition | MyFitnessPal | Mobile Phone | iTunes/GP | 3 undergraduate students | 2019 | 81.7 |
| [16]  Ferrara et al., | Diet, food & nutrition | MyDietCoach | Mobile Phone | iTunes/GP | 3 undergraduate students | 2019 | 46.7 |
| [15]  Bondaronek et al | Physical activity app | Fitbit | Mobile Phone | iTunes/GP | 2 reviewers | 2018 | 66 |
| [15]  Bondaronek et al | Physical activity app | Strava Running and  Cycling GPS | Mobile Phone | iTunes/GP | 2 reviewers | 2018 | 60 |
| [15]  Bondaronek et al | Physical activity app | Pacer – Pedometer plus  Weight Loss and BMI  Tracker | Mobile Phone | iTunes/GP | 2 reviewers | 2018 | 75 |
| [15]  Bondaronek et al | Physical activity app | Map My Run – GPS  Running & Workout  Tracker | Mobile Phone | iTunes/GP | 2 reviewers | 2018 | 85 |
| [15]  Bondaronek et al | Physical activity app | Adidas train & run | Mobile Phone | NA/GP | 2 reviewers | 2018 | 76 |
| [15]  Bondaronek et al | Physical activity app | Steps Pedometer &  Step Counter  Activity Tracker | Mobile Phone | iTunes/NA | 2 reviewers | 2018 | 94 |
| [15]  Bondaronek et al | Physical activity app | 7 Minute Workout  by Simple Design  Ltd | Mobile Phone | NA/GP | 2 reviewers | 2018 | 55 |
| [15]  Bondaronek et al | Physical activity app | Runtastic Running  & Fitness | Mobile Phone | iTunes/GP | 2 reviewers | 2018 | 53 |
| [15]  Bondaronek et al | Physical activity app | Home workout MMA Spartan Free | Mobile Phone | NA/GP | 2 reviewers | 2018 | 91 |
| [15]  Bondaronek et al | Physical activity app | Stepz – Pedometer  & Step Counter | Mobile Phone | iTunes/NA | 2 reviewers | 2018 | 100 |
| [15]  Bondaronek et al | Physical activity app | Interval Timer – Timing for HIIT Training and | Mobile Phone | iTunes/NA | 2 reviewers | 2018 | 85 |
| [15]  Bondaronek et al | Physical activity app | Sworkit – Custom  Workouts for  Exercise & Fitness | Mobile Phone | iTunes/NA | 2 reviewers | 2018 | 96 |
| [15]  Bondaronek et al | Physical activity app | Fitness & Bodybuilding | Mobile Phone | NA/GP | 2 reviewers | 2018 | 80 |
| [15]  Bondaronek et al | Physical activity app | Daily Workouts FREE | Mobile Phone | iTunes/NA | 2 reviewers | 2018 | 71 |
| [15]  Bondaronek et al | Physical activity app | 30 Day Ab Challenge FREE | Mobile Phone | iTunes/GP | 2 reviewers | 2018 | 90 |
| [15]  Bondaronek et al | Physical activity app | Runtastic Results: Body Workout Fitness Trainer | Mobile Phone | iTunes/NA | 2 reviewers | 2018 | 95 |
| [15]  Bondaronek et al | Physical activity app | C25K® - 5K Running  Trainer | Mobile Phone | NA/GP | 2 reviewers | 2018 | 86 |
| [15]  Bondaronek et al | Physical activity app | Health Mate – Steps tracker & Life coach | Mobile Phone | iTunes/NA | 2 reviewers | 2018 | 90 |
| [15]  Bondaronek et al | Physical activity app | One You Couch to  5K | Mobile Phone | iTunes/GP | 2 reviewers | 2018 | 71 |
| [15]  Bondaronek et al | Physical activity app | Running, Walking  and Biking with  Endomondo | Mobile Phone | iTunes/GP | 2 reviewers | 2018 | 86 |
| [15]  Bondaronek et al | Physical activity app | Map My Ride – GPS Cycling & Route Tracker | Mobile Phone | iTunes/GP | 2 reviewers | 2018 | 85 |
| [15]  Bondaronek et al | Physical activity app | Interval Timer | Mobile Phone | NA/GP | 2 reviewers | 2018 | 83 |
| [15]  Bondaronek et al | Physical activity app | 5K Run - Couch to 5K | Mobile Phone | NA/GP | 2 reviewers | 2018 | 91 |
| [15]  Bondaronek et al | Physical activity app | 7 Minutes Workout  – Women Fitness  Exercise Trainer | Mobile Phone | iTunes/NA | 2 reviewers | 2018 | 94 |
| [15]  Bondaronek et al | Physical activity app | Seconds – Interval  Timer | Mobile Phone | iTunes/NA | 2 reviewers | 2018 | 69 |
| [15]  Bondaronek et al | Physical activity app | Running Distance  Tracker + | Mobile Phone | NA/GP | 2 reviewers | 2018 | 91 |
| [15]  Bondaronek et al | Physical activity app | Freeletics  Bodyweight – Workout | Mobile Phone | iTunes/GP | 2 reviewers | 2018 | 84 |
| [15]  Bondaronek et al | Physical activity app | Couch to 10K  Running Trainer | Mobile Phone | NA/GP | 2 reviewers | 2018 | 86 |
| [15]  Bondaronek et al | Physical activity app | FitNotes - Gym  Workout Log | Mobile Phone | NA/GP | 2 reviewers | 2018 | 65 |
| [15]  Bondaronek et al | Physical activity app | Belly Fat Exercises | Mobile Phone | NA/GP | 2 reviewers | 2018 | 73 |
| [15]  Bondaronek et al | Physical activity app | Belly Fat Workout  FREE – 10 Minute  Ab Exercises | Mobile Phone | iTunes/NA | 2 reviewers | 2018 | 75 |
| [15]  Bondaronek et al | Physical activity app | Movesum – Step  counter by Lifesum | Mobile Phone | iTunes/NA | 2 reviewers | 2018 | 99 |
| [15]  Bondaronek et al | Physical activity app | 7 Minute Workout  Challenge by  Fitness Guide Inc | Mobile Phone | iTunes/GP | 2 reviewers | 2018 | 100 |
| [15]  Bondaronek et al | Physical activity app | Adrian James 6  Pack Abs Workout | Mobile Phone | iTunes/NA | 2 reviewers | 2018 | 99 |
| [15]  Bondaronek et al | Physical activity app | Full Fitness :  Exercise Workout  Trainer | Mobile Phone | iTunes/NA | 2 reviewers | 2018 | 61 |
| [15]  Bondaronek et al | Physical activity app | Runtastic PRO  Running and  Workout Tracker | Mobile Phone | iTunes/GP | 2 reviewers | 2018 | 95 |
| [15]  Bondaronek et al | Physical activity app | Couch to 5K® - Running App and Training Coach | Mobile Phone | iTunes/GP | 2 reviewers | 2018 | 78 |
| [15]  Bondaronek et al | Physical activity app | Adrian James High Intensity Interval Training | Mobile Phone | iTunes/NA | 2 reviewers | 2018 | 99 |
| [15]  Bondaronek et al | Physical activity app | Running for Weight Loss PRO | Mobile Phone | iTunes/NA | 2 reviewers | 2018 | 91 |
| [15]  Bondaronek et al | Physical activity app | Fitness Trainer FULL version | Mobile Phone | NA/GP | 2 reviewers | 2018 | 78 |
| [15]  Bondaronek et al | Physical activity app | Instant Fitness :  600+ exercises,  100+ workouts… | Mobile Phone | iTunes/NA | 2 reviewers | 2018 | 89 |
| [15]  Bondaronek et al | Physical activity app | Push ups 0 to 100: push ups challenge trainer pro | Mobile Phone | iTunes/NA | 2 reviewers | 2018 | 99 |
| [15]  Bondaronek et al | Physical activity app | Couch to 5K Runner, 0 to 5K run training | Mobile Phone | iTunes/NA | 2 reviewers | 2018 | 99 |
| [15]  Bondaronek et al | Physical activity app | Footsteps – Pedometer | Mobile Phone | iTunes/NA | 2 reviewers | 2018 | 61 |
| [15]  Bondaronek et al | Physical activity app | iMuscle 2 | Mobile Phone | iTunes/GP | 2 reviewers | 2018 | 66 |
| [15]  Bondaronek et al | Physical activity app | 10K Running Trainer Pro | Mobile Phone | NA/GP | 2 reviewers | 2018 | 88 |
| [15]  Bondaronek et al | Physical activity app | Police Fitness – Bleep Test | Mobile Phone | iTunes/NA | 2 reviewers | 2018 | 80 |
| [15]  Bondaronek et al | Physical activity app | Marathon Trainer - 26.2 42K | Mobile Phone | NA/GP | 2 reviewers | 2018 | 88 |
| [15]  Bondaronek et al | Physical activity app | MapMyFitness+ Workout Trainer | Mobile Phone | NA/GP | 2 reviewers | 2018 | 93 |
| [15]  Bondaronek et al | Physical activity app | 5K to 10K | Mobile Phone | iTunes/GP | 2 reviewers | 2018 | 88 |
| [15]  Bondaronek et al | Physical activity app | 7 Minute Workout Pro | Mobile Phone | NA/GP | 2 reviewers | 2018 | 85 |
| [15]  Bondaronek et al | Physical activity app | Chloe Madeley Weights 4 Women | Mobile Phone | iTunes/GP | 2 reviewers | 2018 | 84 |
| [15]  Bondaronek et al | Physical activity app | 10K Pacer: Run pace training. Run faster | Mobile Phone | iTunes/NA | 2 reviewers | 2018 | 99 |
| [15]  Bondaronek et al | Physical activity app | Starting Strength Official | Mobile Phone | NA/GP | 2 reviewers | 2018 | 58 |
| [15]  Bondaronek et al | Physical activity app | Thor Fitness: 60 Day Bodyweight Workout Routine | Mobile Phone | iTunes/NA | 2 reviewers | 2018 | 69 |
| [15]  Bondaronek et al | Physical activity app | Half Marathon Trainer 13.1 21K | Mobile Phone | iTunes/NA | 2 reviewers | 2018 | 85 |
| [15]  Bondaronek et al | Physical activity app | Yoga Break | Mobile Phone | iTunes/NA | 2 reviewers | 2018 | 93 |
| [15]  Bondaronek et al | Physical activity app | PDC Pole Dance Syllabus | Mobile Phone | NA/GP | 2 reviewers | 2018 | 83 |
| [15]  Bondaronek et al | Physical activity app | MMA Spartan Workouts Pro | Mobile Phone | NA/GP | 2 reviewers | 2018 | 90 |
| [15]  Bondaronek et al | Physical activity app | Get Running (Coach to 5K) | Mobile Phone | iTunes/NA | 2 reviewers | 2018 | 88 |
| [15]  Bondaronek et al | Physical activity app | WalkJogRun GPS Running Routes | Mobile Phone | iTunes/NA | 2 reviewers | 2018 | 71 |
| [15]  Bondaronek et al | Physical activity app | Runtastic Road Bike PRO | Mobile Phone | NA/GP | 2 reviewers | 2018 | 91 |
| [15]  Bondaronek et al | Physical activity app | Runtastic Mountain Bike PRO GPS Biking Computer, | Mobile Phone | iTunes/GP | 2 reviewers | 2018 | 94 |
| [15]  Bondaronek et al | Physical activity app | Chloe Madeley 15 minute fat loss workout | Mobile Phone | iTunes/NA | 2 reviewers | 2018 | 90 |
| [15]  Bondaronek et al | Physical activity app | CARROT Fit – 7 Minute Workout, Step Counter Weight Tracker | Mobile Phone | iTunes/NA | 2 reviewers | 2018 | 94 |
| [17]  Isaković et al., | Diabetes monitoring | DeStress Assistant (DeSA) | Mobile Phone |  |  | 2016 | 84.5 |
| [18]  Metelmann et al | First aid, CPR and/or choking | HELP Notfall | Mobile Phone | iTunes/GP | 9 emergency physicians + 10 frequent mobile phone users | 2018 | 87.5 |
| [18]  Metelmann et al | First aid, CPR and/or choking | HAMBURG SCHOCKT | Mobile Phone | iTunes/GP | 9 emergency physicians + 10 frequent mobile phone users | 2018 | 55 |
| [18]  Metelmann et al | First aid, CPR and/or choking | Mein DRK | Mobile Phone | iTunes/GP | 9 emergency physicians + 10 frequent mobile phone users | 2018 | 32.5 |
| [19]  Gowarty et al., | Smoking cessation | QuitGuide | Mobile Phone | iTunes/GP | 17 smokers | 2021 | 66 |
| [19]  Gowarty et al., | Smoking cessation | quitSTART | Mobile Phone | iTunes/GP | 17 smokers | 2021 | 64 |
| [20]  Morey et al., | Managing heart failure | Heart Partner | Mobile Phone | iTunes | 6 adults (ages 65 to 85) | 2017 | 42.5 |
| [20]  Morey et al., | Managing heart failure | Heart Failure Health Storylines | Mobile Phone | iTunes | 6 adults (ages 65 to 85) | 2017 | 60 |
| [5]  Islam et al., | General health informative app | Patient Aid | Mobile Phone |  | 10 evaluators | 2020 | 65.7 |
| [5]  Islam et al., | Physician information | DIMS | Mobile Phone |  | 10 evaluators | 2020 | 73.2 |
| [5]  Islam et al., | Institutional app | Hospital Finder | Mobile Phone |  | 10 evaluators | 2020 | 52.8 |
| [5]  Islam et al., | Physical activity app | Bangla Gym Guide | Mobile Phone |  | 10 evaluators | 2020 | 70.5 |
| [5]  Islam et al., | Mother and child (pregnancy and new mothers support) | Aponjon Pregnancy | Mobile Phone |  | 10 evaluators | 2020 | 79.2 |
| [5]  Islam et al., | Disease specific care app | এলার্জরি সহজ চিচিৎসা <Easy Treatment for Allergy> | Mobile Phone |  | 10 evaluators | 2020 | 69.3 |
| [5]  Islam et al., | Herbology | ভেষজ চিচিৎসা <Herbal Treatment> | Mobile Phone |  | 10 evaluators | 2020 | 68.3 |
| [5]  Islam et al., | Diet, food & nutrition | Fruits Benefit in Bangla | Mobile Phone |  | 10 evaluators | 2020 | 71.7 |
| [5]  Islam et al., | Homeopathic | Homeopathic Bangla book | Mobile Phone |  | 10 evaluators | 2020 | 65.4 |
| [21]  Gibson et al., | Personalised reminiscence for people living with dementia. | InspireD | Tablet | iTunes | 7 caregivers | 2016 | 67.5 |
| [21]  Gibson et al., | Personalised reminiscence for people living with dementia. | InspireD | Tablet | iTunes | 4 people living with dementia | 2016 | 78.75 |
| [22]  O’Grady et al., | Suicide prevention | SafePlan | Mobile Phone |  |  | 2020 | 71.81 |
| [23]  Browne et al., | Pediatric obesity treatment | Mandolean | Mobile Phone | iTunes/GP | 20 participants | 2020 | 54.1 |
| [23]  Browne et al., | Pediatric obesity treatment | myBigO | smart watch | iTunes/GP | 20 participants | 2020 | 75.9 |
| [24]  Teixeira et al., | Voice health | Unknown | Unknown | Unknown | 26 participants | 2019 | 87.3 |
| [25]  Kalz et al., | First aid, CPR and/or choking | Reanimatie (Dutch) | Mobile Phone | iTunes/GP | 14 volunteers | 2014 | 82 |
| [25]  Kalz et al., | First aid, CPR and/or choking | CPR & Choking (English) | Mobile Phone | iTunes/GP | 14 volunteers | 2014 | 73 |
| [25]  Kalz et al., | First aid, CPR and/or choking | FDNY Lifesaver Beta V1.0 (English) | Mobile Phone | iTunes/GP | 14 volunteers | 2014 | 72 |
| [25]  Kalz et al., | First aid, CPR and/or choking | Leben retten (German) | Mobile Phone | iTunes/GP | 14 volunteers | 2014 | 70.5 |
| [25]  Kalz et al., | First aid, CPR and/or choking | Hands-Only CPR (English) | Mobile Phone | iTunes/GP | 14 volunteers | 2014 | 68.5 |
| [25]  Kalz et al., | First aid, CPR and/or choking | St. John Ambulance First Aid (English) | Mobile Phone | iTunes/GP | 14 volunteers | 2014 | 67 |
| [25]  Kalz et al., | First aid, CPR and/or choking | Emergency First Aid & Treatment Guide (English) | Mobile Phone | iTunes/GP | 14 volunteers | 2014 | 61.9 |
| [25]  Kalz et al., | First aid, CPR and/or choking | Free CPR (aka CPR Steps) (English) | Mobile Phone | iTunes/GP | 14 volunteers | 2014 | 61.5 |
| [25]  Kalz et al., | First aid, CPR and/or choking | SOS American Red Cross (English) | Mobile Phone | iTunes/GP | 14 volunteers | 2014 | 61.5 |
| [25]  Kalz et al., | First aid, CPR and/or choking | PocketCPR (English) | Mobile Phone | iTunes/GP | 14 volunteers | 2014 | 53.8 |
| [25]  Kalz et al., | First aid, CPR and/or choking | Pocket First Aid & CPR (English) | Mobile Phone | iTunes/GP | 14 volunteers | 2014 | 52 |
| [25]  Kalz et al., | First aid, CPR and/or choking | First Aid White Cross (English) | Mobile Phone | iTunes/GP | 14 volunteers | 2014 | 45.5 |
| [25]  Kalz et al., | First aid, CPR and/or choking | SCDF Choking CPR AED (English) | Mobile Phone | iTunes/GP | 14 volunteers | 2014 | 36.5 |
| [26]  Banos et al., | Support trunk endurance assessment | mDurance | Mobile Phone + Wearable sensors | GP | 7 evaluators | 2015 | 84.29 |
| [27]  Goldsmith et al., | Health communication | Health Communication iOS app | Mobile Phone | iTunes | 8 participants | 2015 | 91.56 |
| [28]  KIZAKEVICH et al., | Health intervention (self-assessment, self-help) | Personal Health Intervention Toolkit (PHIT) | Mobile Phone |  | 31 participants | 2014 | 85 |
| [29]  Hoevenaars et al., | Promote a healthy lifestyle in wheelchair users with spinal cord injury or lower limb amputation | WHEELS | Mobile Phone |  | 14 participants | 2021 | 58.6 |
| [30]  Fuller-Tyszkiewicz et al., | Intervention for depression | BlueWatch | Mobile Phone |  | 5 participants | 2018 | 86 |
| [30]  Fuller-Tyszkiewicz et al., | Intervention for depression | BlueWatch | Mobile Phone |  | 5 researchers | 2018 | 60.5 |
| [30]  Fuller-Tyszkiewicz et al., | Intervention for depression | BlueWatch | Mobile Phone |  | 5 clinicians | 2018 | 67 |
| [31]  Salamah et al., | Personal health record for people with autoimmune disease | Thymun | Mobile Phone |  | 5 participants | 2021 | 74 |
| [32]  Cameron et al., | Mental health | iHelpr | Mobile Phone |  | 7 participants | 2019 | 88.2 |
